# Quality of life in women with endometriosis: a cross-sectional survey

**DOI:** 10.1007/s11136-020-02515-4

**Published:** 2020-04-30

**Authors:** Agnieszka Bień, Ewa Rzońca, Marta Zarajczyk, Katarzyna Wilkosz, Artur Wdowiak, Grażyna Iwanowicz-Palus

**Affiliations:** 1 Department of Development in Midwifery, Faculty of Health Sciences, Medical University of Lublin, Lublin, Poland; 2 Department of Development in Midwifery, Medical University, Lublin, Poland; 3 Diagnostic Techniques Unit, Faculty of Health Sciences, Medical University of Lublin, Lublin, Poland

**Keywords:** Quality of life, Women, Endometriosis, Acceptance of illness

## Abstract

**Purpose:**

The aim of the study was to assess QoL and identify and analyse its determinants in women with endometriosis.

**Methods:**

The study was performed in 2019 in health centres in Lublin (Poland) on 309 women with diagnosed endometriosis. In order to verify which factors affect QoL of the study participants, regression for qualitative variables (CATREG) was used. The applied research instruments included the WHOQOL-BREF quality of life questionnaire, the Acceptance of Illness Scale (AIS), the Laitinen Pain Scale, and a general questionnaire.

**Results:**

The overall QoL score of the respondents was 3.30, whereas their overall perceived health score was 2.37. The highest QoL scores were found for the psychological domain 13.33, whereas the lowest QoL were found for the physical domain 11.52. Women with endometriosis have a moderate level of illness acceptance (24.64) and experience daily pain of moderate intensity (5.83).

**Conclusion:**

Women with endometriosis rate their overall QoL higher than their overall perceived health. Perceived QoL in women with endometriosis is most commonly associated with their acceptance of illness, BMI, negative impact of symptoms on the relationship with the partner, and dyspareunia. To improve these women’s lives, care should also respond to the social, emotional, and sexual issues resulting from the illness. Such interventions will contribute to improved comfort and QoL among these women.

## Introduction

Endometriosis is a chronic condition in which the endometrial cells lining the body of the uterus, which show secretory activity, are found outside the uterus. The cells react to the hormonal changes that occur during the menstrual cycle, which leads to chronic inflammation. The symptoms of endometriosis are similar to those of other conditions and include severe pain during menstrual periods and during sexual intercourse, pain in lower abdomen (sometimes in the sacral region), and pain during urination and during a gynaecological examination [1–3]. It is estimated that endometriosis affects between 7 and 15% of women of childbearing age, including between 30 and 50% of infertile women and almost 50% of women with chronic pelvic pain syndrome. These are only estimates, as endometriosis may be asymptomatic, which makes it impossible to determine the exact number of women suffering from the condition [4–6].

The literature on the subject frequently draws attention to the impact of endometriosis on different areas of life. Given the symptoms and complications associated with endometriosis, particular attention should be paid not only to the physical condition of a given patient but also to the social aspects and psychological consequences of being diagnosed with the condition. Similarly, the effectiveness of treatment should be evaluated based not only on the clinical and functional assessment of a given patient but also on the assessment of her quality of life (QoL) [5, 7, 8].

The assessment of QoL is aimed at choosing the optimum treatment and care option for a patient, taking into account their health status and their physical, mental, and social well-being [7, 9]. The WHO defines QoL as an individual's perception of their position in life in the context of the culture and value systems in which they live and in relation to their goals, expectations, standards, and concerns, as determined by environmental conditions [10]. QoL indicators include the ability to continue to fulfil one's social roles, the ability to adjust, mental well-being, and functioning in social groups [7, 10].

Due to its prevalence and its social and economic implications, endometriosis is of particular interest to researchers studying QoL. The assessment and perception of QoL are subjective and individual and depend on many factors, both dependent on and independent of a patient. It is currently believed that the assessment of QoL is an important element of the medical care of a patient, as it does not focus on treating a given illness but rather on the symptoms experienced by the patient and their impact on the patient’s life [7, 8].

Acceptance of illness is a major factor in QoL evaluation, and adaptation to illness (or lack thereof) affects both patients’ QoL and their satisfaction with life. Acceptance of illness evaluation is consistent with the increasing focus on QoL in medical science, resulting from changes in care models that increasingly emphasize the need for a comprehensive assessment of a patient’s health, including a reference to their living standard and social position in their environment. Multiple studies have demonstrated a positive correlation between acceptance of illness and QoL in chronically ill patients, but little is yet known about the relationship between acceptance of illness and QoL in women with endometriosis [11–13].

In the light of the above, the aim of the study was to assess QoL and identify and analyse its determinants in women with endometriosis.

## Method

### Study population and recruitment

The study was performed in 2019 in 3 health centres in Lublin (Poland) on 309 women with diagnosed endometriosis. The study included women who reported to the health centres due to complaints associated with their illness or for follow-up, e.g. for a cervical smear. All women included in the study were Caucasian, spoke and understood Polish, and were aged between 18 and 47 years.

The criteria for inclusion in the study were as follows: use of healthcare services in Lublin Province, histologic and surgical confirmation of endometriosis (by laparoscopy or laparotomy), diagnosis of stage 3 or stage 4 endometriosis in accordance with the guidelines of the American Society for Reproductive Medicine within the last 2 years [14].

Patients suffering from any other chronic illness or cancer, patients with psychological disorders, and patients who had undergone surgery on the abdomen (other than surgery to confirm the diagnosis of endometriosis), chest, or spine were excluded from the study. Age below 18 or above 46 years and post-menopausal status were also exclusion criteria.

The standardized interview questionnaire used to collect data on respondents’ characteristics included items on their age (mean age was calculated), education (2 categories: “lower than university” and “university”), relationship status (married or “other”, the latter including other types of relationships), perceived family wealth (very wealthy/wealthy, average, rather poor/poor), and BMI.

**Fig. 1:**
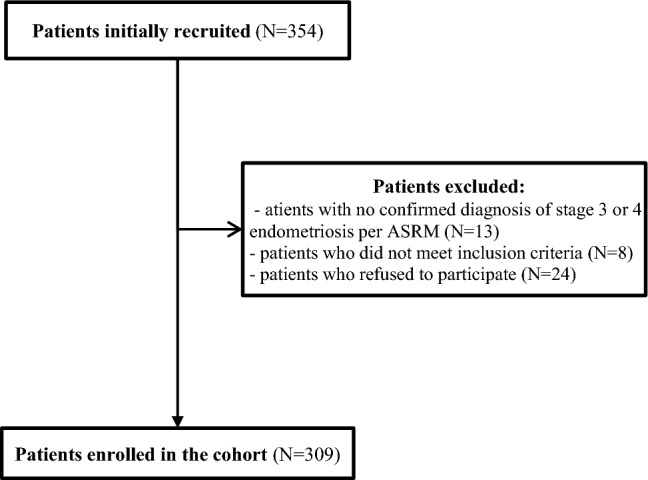
Flowchart of the recruitment process of the patients

Each study participant was informed about the aim of the study and provided with instructions on how to answer the questions. All participants completed the questionnaire by themselves, with no assistance. 354 surveys were distributed, and 309 correctly completed surveys were returned. 13 patients were not included because they did not meet the endometriosis stage criterion, 8 did not meet other inclusion criteria, and 24 refused to participate (Fig. 1). Survey completion time was approximately 15 min. Return rate was 87.28%.

### Ethics

The study was approved by the Bioethics Committee of the Medical University of Lublin (KE-0254/200/2019). The study participants were informed that participation in the study was voluntary and anonymous, and that the results obtained would only be used for scientific purposes.

### Measures

The research tools used were as follows: WHOQOL-BREF quality of life questionnaire, Acceptance of Illness Scale (AIS), Laitinen scale for the assessment of pain, and a standardized interview questionnaire including questions regarding the characteristics of the women participating in the study.

The WHOQOL-BREF (*World Health Organization Quality of Life-Bref*) questionnaire is a multicultural tool designed for the assessment of QoL in four domains: physical, psychological, social relationships and environment, and a patient’s overall perception of their QoL and health. The questionnaire is a set of 26 questions scored on a scale from 0 to 5. The arithmetic mean score of items within each domain is used to calculate the domain score. Scores for overall perceived QoL and overall perceived health as well as domain scores are scaled in a positive direction, which means that higher scores denote a higher QoL. The Cronbach's alpha coefficient is as follows: between 0.54 and 0.91 for particular dimensions and 0.92 (healthy respondents) and 0.95 (respondents with illness) for the entire scale [10]. The reliability of the questionnaire in the study was high and was as follows: 0.830 for the physical domain, 0.830 for the psychological domain, 0.735 for the social relationships domain, and 0.792 for the environment domain.

The Acceptance of Illness Scale (*AIS*) is designed to assess the level of acceptance of illness in adults and can be applied for each disease entity. The AIS consists of 8 statements concerning the negative consequences of illness rated on a five-point scale where *1* means *strongly agree* and *5* means *strongly disagree.* The level of acceptance of illness is the sum of the points scored (from 8 to 40). The higher the score, the better a patient is coping with the limitations caused by their illness and the lower the level of the psychological discomfort associated with the illness. A score of below 20 is considered low and indicates a lack of or a low level of acceptance of and adaptation to illness, as well as significant emotional problems associated with the illness. A score of between 20 and 30 means a moderate level of acceptance of illness. The scale’s internal consistency estimated using Cronbach’s alpha was 0.85; the reliability of the Polish acceptance of illness scale is similar to that of the original version, with Cronbach’s alpha of 0.82. Cronbach’s alpha in our own study was 0.898 [15].

*The Laitinen Pain Scale* is a tool for the subjective assessment of pain. It consists of four questions in which patients rate the intensity and frequency of pain, the frequency of analgesics use, and physical activity limitations. Each question is scored from 0 to 4, where 0 means ‘no problem’ and 4 means ‘the biggest possible problem’ [16]. The reliability of the questionnaire in the study, measured by Cronbach's alpha, was 0.820.

### Statistical analysis

The research material collected was analysed statistically using Statistica v.12.5 software. The values of the quantitative parameters analysed were presented as mean (M), standard deviation (SD), and median (Me), whereas qualitative parameters were presented as number or percentage values. In order to verify which factors affect QoL of the study participants, regression for qualitative variables (CATREG—Categorical Regression using Alternating Least Squares) was used. The CATREG technique complements the standard method with simultaneous scaling of nominal, ordinal, and numerical variables. It quantifies qualitative variables so as to reflect the characteristics of the original categories. Quantified qualitative variables are then treated the same way as numerical variables. The use of non-linear transformations allows for analysing variables on different levels to find the best-fitting model. The level of significance used in the study was *p* < 0.05.

## Results

Table 1 shows the characteristics of the women participating in the study: mean age was 30.86 years, the largest group of respondents had higher education (70.55%), were married (64.08%), perceived the financial situation of their families as average (84.14%), considered endometriosis treatment expensive (92.23%), and had a BMI within the normal range (60.52%). Out of the 295 respondents who were in a relationship, 72.20% reported that their illness had a negative impact on their relationship with the partner. The majority of the respondents stated that the symptoms of their condition disrupt their day-to-day functioning (63.43%) and that they experience dyspareunia during sexual intercourse (73.14%). As many as 93.20% of the respondents had painful periods and 85.76% of the respondents suffering from painful periods reported that the pain disrupted their day-to-day functioning.

**Table 1:** Participants’ characteristics

Participants’ characteristics	*N*	%
Mean age (SD)	30.86 (± 5.57), range 18–46	
Education		
Lower than university	91	29.45
University	218	70.55
Relationship status		
Other	111	35.92
Married	198	64.08
Perceived family wealth		
Very wealthy/rather wealthy	34	11.00
Average	260	84.14
Rather poor/poor	15	4.86
Is endometriosis treatment expensive		
Yes	285	92.23
No	24	7.77
BMI		
Underweight (< 18.50)	38	12.30
Normal (18.50–24.99)	187	60.52
Overweight (25.00–29.99)	50	16.18
Obese (> 30.00)	34	11.00
Impact of the condition on the relationship with the partner (*N* = 295)		
Yes	213	72.20
No	82	27.80
Symptoms of the condition disrupt day-to-day functioning		
Yes	196	63.43
No	113	36.57
Dyspareunia		
Yes	226	73.14
No	83	26.86
Painful periods		
Yes	288	93.20
No	21	6.80
Menstrual pain disrupts day-to-day functioning (*N* = 288)		
Yes	247	85.76
No	41	14.24

The overall QoL score among the respondents was 3.30 ± 0.91, whereas their overall perceived health score was 2.37 ± 0.93. The highest QoL scores were found for the psychological domain—13.33 ± 2.28, whereas the lowest QoL scores were found for the physical domain—11.52 ± 3.02 (Table 2).

**Table 2:** Quality of life of women with endometriosis

Domains	*M*	Me	SD
General quality of life	3.30	3.00	0.91
General health	2.37	2.00	0.93
Physical health	11.52	11.43	3.02
Psychological	13.33	13.33	2.28
Social relationships	12.90	13.33	3.51
Environment	12.85	13.00	2.68

*M* mean, *Me* median, *SD* standard deviation

The mean Acceptance of Illness Scale (AIS) score of the respondents with endometriosis was 24.64 ± 8.60, whereas the mean Laitinen pain scale score was 5.83 ± 3.30 (Table 3).

**Table 3:** Mean pain intensity and acceptance of illness scores in women with endometriosis

Statement	Score		
	*M*	Me	SD
Mean acceptance of illness score	24.64	25.00	8.60
Mean pain intensity score	5.83	5.00	3.30

*M* mean, *Me* median, *SD* standard deviation

The scaling of multi-level variables showed that lower QoL scores in the physical domain were significantly associated with pain intensity, acceptance of illness, impact of the illness on relationship with the partner, and education (the higher, the better the score). Lower QoL scores in the psychological domain are correlated with the following: acceptance of illness, BMI (obesity), the impact of the pain experienced during menstrual periods on day-to-day functioning, and dyspareunia. For the social relationships domain, statistically significant predictors were as follows: acceptance of illness, dyspareunia, impact of symptoms on the relationship with the partner, and age. QoL in the environment domain was then analysed. The analysis identified the following significant predictors of scores in the environment domain: acceptance of illness, BMI (obesity), cost of endometriosis treatment, and family wealth (rather poor/poor) (Table 4).

**Table 4:** Regression analysis for WHOQOL domains in women with endometriosis

Predictors	Physical health *F*(16.208) = 15.348; *p* < 0,001; *R*^2^ = 0.506			
	*β*	SE	*F*	*p*
Pain intensity score	− 0.322	0.068	22.226	0.000
Acceptance of illness (AIS)	0.316	0.061	26.349	0.000
Endometriosis has an impact on the relationship with a partner	0.159	0.056	7.979	0.005
Education	0.099	0.049	4.112	0.044

Predictors	Psychological *F*(16.208) = 7.258; *p* < 0.001; *R*^2^ = 0.309			
	*β*	SE	*F*	*p*
Acceptance of illness (AIS)	0.348	0.073	22.826	0.000
BMI	− 0.249	0.065	14.685	0.000
Menstrual pain disrupts day-to-day functioning	0.174	0.068	6.493	0.012
Dyspareunia	0.168	0.072	5.487	0.020

Predictors	Social relationship *F*(16.208) = 6.021; *p* < 0.001; *R*^2^ = 0.264			
	*β*	SE	*F*	*p*
Acceptance of illness (AIS)	0.298	0.100	8.904	0.003
Dyspareunia	0.225	0.076	8.834	0.003
Endometriosis has an impact on the relationship with the partner	0.223	0.084	7.050	0.009
Age	− 0.197	0.076	6.663	0.011

Predictors	Environmental *F*(16.208) = 4.161; *p* < 0.001; *R*^2^ = 0.184			
	*β*	SE	*F*	*p*
Acceptance of illness (AIS)	0.259	0.075	12.018	0.001
BMI	− 0.170	0.061	7.880	0.005
Cost of endometriosis treatment	0.130	0.048	7.337	0.007
Perceived family wealth	0.123	0.065	3.601	0.029

*Β* standardized coefficients, *SE* bootstrapped standard errors, *BMI* Body Mass Index

## Discussion

Endometriosis is a chronic condition which affects an increasing number of women. The literature on the subject emphasizes its impact on different areas of life. In numerous women, endometriosis causes infertility and chronic pain and has a negative impact on well-being. It also places a significant financial burden on the healthcare system [5, 8, 17–19]. As the assessment of QoL is one of the methods used to assess the consequences of chronic conditions and is a functional indicator of the effectiveness of treatment, the aim of the present study was to assess the level of and identify and analyse the determinants of QoL in women with endometriosis.

Our study found that the overall QoL of the study participants with endometriosis was significantly higher compared with their overall perceived health. A study by Nnoaham et al. on QoL of women with endometriosis showed that QoL of women diagnosed with endometriosis was lower than that of women who had similar symptoms but who were not diagnosed with endometriosis [20]. Similarly, Facchin et al. found that QoL of women with endometriosis and pelvic pain was lower compared with patients with asymptomatic endometriosis and women in the control group [21]. In turn, Soliman et al. showed that the greater the number and intensity of the symptoms of endometriosis, the lower the HRQoL of women diagnosed with the condition [6]. In the present study, the lowest QoL scores in women with endometriosis were found for the physical domain. Those results show that the main problem for the respondents is pain, the necessity to take medication regularly in order to function “normally”, and also the lack of satisfaction with their performance in everyday life and their work capacity.

One other important aspect analysed in the present study was acceptance of illness by the study participants with diagnosed endometriosis. Acceptance of illness is a significant element of the process of adjusting to a life with illness and is associated with the severity of the negative emotional reactions caused by the illness. The higher the level of acceptance of somatic illness, the higher the level of adjustment to the illness and the lower the discomfort it causes, which, in practice, means maintained self-esteem and less stress for the patient [22]. The mean acceptance of illness score obtained by the study participants with endometriosis was 24.64, which denotes a moderate level of acceptance of illness. Those results are consistent with the findings by other authors analysing the level of acceptance of illness in patients with a chronic condition [23–25]. The moderate acceptance of illness scores may suggest that the study participants have partially adjusted to their illness and its negative implications. Patients who accept their illness take an optimistic attitude to the condition and understand it better. They also trust their physicians, have confidence in the treatment received, and actively participate in the therapy process [22].

The literature on the subject also discusses the issue of the relationship between the patients' acceptance of illness and QoL. Jankowska-Polańska et al. showed that the higher the acceptance of illness scores in patients with chronic obstructive pulmonary disease, the higher their QoL [26]. Szpilewska et al. and Bień et al., who studied patients with a stoma and pregnant women with diabetes, respectively, obtained similar results [12, 23]. Acceptance of illness means taking a positive approach to the illness. It helps mobilize one’s resources and prevent a decrease in QoL as a result of chronic illness [12, 23, 26]. Those findings are consistent with the results of our study.

In women with endometriosis, age is a factor associated with QoL in the social relationships domain. Older respondents have a lower QoL in this aspect. Moradi et al. demonstrated a number of similarities and differences between patients of different ages. Similarities independent of age included marital/sexual relationships, social life, and physical and psychological consequences of the illness, while differences were found in terms of education—for women under 24, opportunities and employment—for women aged between 25 and 34, and financial standing for women older than 35 [8].

The present analyses revealed that there is a correlation between the level of education and QoL of women with endometriosis. Women with higher education had higher QoL scores in the physical domain. They are able to manage their condition better, and are aware of the need for treatment with a number of specialists, including nutritionists, physical therapists, pain management specialists, psychologist, and others. Those findings are consistent with the results obtained by Shum et al. [27].

One of the factors that may hinder coping with a chronic condition is the lack of sufficient financial resources. Financial resources are an important factor as regards the treatment of endometriosis, as patients with the condition have to pay for medication, fertility treatment and complementary therapies (acupuncture, yoga, physiotherapy, psychotherapy), and even sanitary towels [18, 28, 29]. Other significant problems include the need to switch to part-time work, reduced income as a result of the lack of paid sick leave, or the inability to take up a holiday job as a result of surgery scheduled for a holiday period [8]. The present analyses demonstrated that family wealth and expense associated with treatment were associated with QoL in the environmental aspect. A lower QoL in the environment domain means, among other things, a lower level of satisfaction with one’s performance in everyday life, work capacity, and personal relations with others.

Our study found that the QoL scores of the respondents in the psychological and environment domains were correlated with their BMI. Women with obesity had the lowest QoL scores in those domains. It was found that they enjoy their lives less and are less satisfied with themselves, their intimate lives, and their appearance. Those results differ from the results obtained by Gallagher et al., who showed that QoL of underweight women was lower compared with women with normal weight [9].

In the present study, most respondents felt their symptoms had an adverse impact on their relationship with the partner. These findings are consistent with the results obtained by Pluchino et al. [30]. De Graaff et al. stress that the condition may even be a cause of divorce [18]. A difficult relationship with a partner results in a lower QoL in the physical and social relationships domains. The factors which have a significant impact on the relationship that women with endometriosis have with their partner include not only the symptoms of the condition, which affect different areas of their daily life, but also the pain they experience during sexual intercourse, as a result of which they often avoid sexual intercourse or significantly limit sexual activity [31, 32].

In the present study, almost three-quarters of the participants with endometriosis experienced pain during sexual intercourse. Those women had lower QoL scores in the psychological and social relationships domains. The pain that women with endometriosis experience during sexual intercourse has an impact on their QoL and results in decreased libido, pelvic discomfort, and less intense and less satisfying orgasms. Women with endometriosis are less relaxed and satisfied after sexual intercourse [27, 32–34]. Dyspareunia may also have an impact on the sexual function of partners, e.g. on erectile dysfunction [30].

Pain is among the most common complaints among patients with endometriosis [2, 3, 6]. The respondents rated the intensity of the pain they experience every day as moderate. Numerous studies have shown clearly that pain has the most negative impact on how women with endometriosis function [33, 35–38]. According to Melis et al., pain reduction is of key importance, as pain is the most significant factor affecting QoL parameters in women with diagnosed endometriosis [33].

Souza et al. found that the greater the intensity of pelvic pain in women with endometriosis, the lower their perceived QoL in the psychological and environment domains [37]. This is consistent with the results obtained by McPeak et al., who found that women with endometriosis rate their QoL lower as a result of the pain associated with the condition [38]. In turn, a study by Giuliani et al. did not confirm the hypothesis that increased pain intensity results in a lower overall QoL [39].

The chronic pain associated with endometriosis may contribute to lower productivity in female workers suffering from the condition. One additional problem is the fact that employers tend to ignore the symptoms of the condition. Fourquet et al. found that some of the women participating in their study had to reduce their working hours or change jobs as a result of the symptoms of the condition, which made it more difficult for them to work [19].

Our study showed that one of the predictors of lower QoL scores in the physical domain in women with endometriosis is menstrual pain, which disrupts their day-to-day functioning. The negative impact of painful periods on QoL of women with endometriosis was confirmed by Altinbas et al., who found that painful periods were negatively correlated with QoL scores in the physical, social relationships and environment domains [40]. In turn, an international multi-centre cross-sectional study found that dysmenorrhoea was not a significant factor in the multivariate analysis, showing no independent effect on either the physical or the mental component [18].

### Strengths and limitations of this study

The present study is one of the few to date that analysed QoL, acceptance of illness, and pain intensity in women with endometriosis. Confirmation of endometriosis severity ensures high quality of data.

One of its limitations lies in its cross-sectional design, which does not allow for identifying any causal relationship between endometriosis and QoL. Another is due to the fact that its design did not include patients’ gynaecological history, e.g. their menstrual cycles, reproductive health, pregnancies, and births. Notably, the regression analysis method used for qualitative variables (CATREG) enabled the identification of variables associated with high and low QoL in the patients studied. The present study may be a preliminary to future prospective, longitudinal studies on the impact of endometriosis on QoL.

### Study implications and conclusion

Research on QoL helps identify threats and individualize the treatment and care process and provides indicators for planning and providing holistic care to individual patients or groups of patients. Studies on patients’ subjectively perceived health may have an important cognitive, practical, and prognostic value and may thus help improve the quality of care of patients with diagnosed endometriosis.

The present study on QoL in women with endometriosis shows variability in factors, indicating the need for a multidisciplinary, individual approach to a patient. As stressed by Rowe et al., patient-oriented care, characterized by continuity, respect, and provision of information, may improve HRQoL in women with endometriosis [41]. The results of the study may help identify those areas of life of patients with endometriosis which are the most problematic as a result of the condition.

Women with endometriosis rate their overall QoL higher than their overall perceived health. Factors that affect the perceived QoL in women with endometriosis include acceptance of illness (AIS score), pain intensity, BMI, negative impact of symptoms on the relationship with the partner, dyspareunia, expensive endometriosis treatment, age, pain experienced during menstrual periods, family wealth, and education.

The present study suggests the need for raising awareness on endometriosis in the society and distributing reliable knowledge on its symptoms and impact on the daily lives of affected women. This could help in early diagnosis and treatment, and prevent misunderstanding associated with prolonged diagnostics. To improve these women’s lives, care should go beyond focusing on symptoms and their impact on daily life, and respond to the social, emotional, and sexual issues resulting from the illness. Such interventions will contribute to improved comfort and QoL among women with endometriosis.
